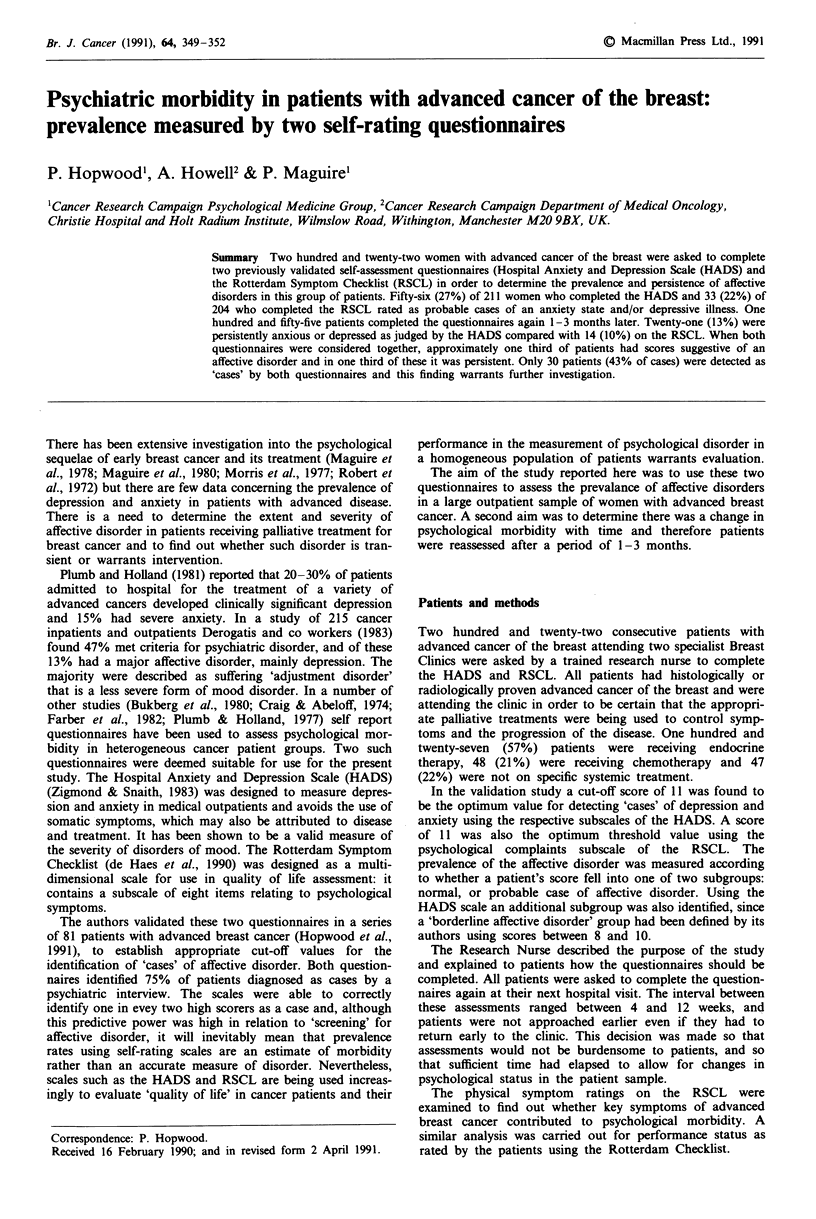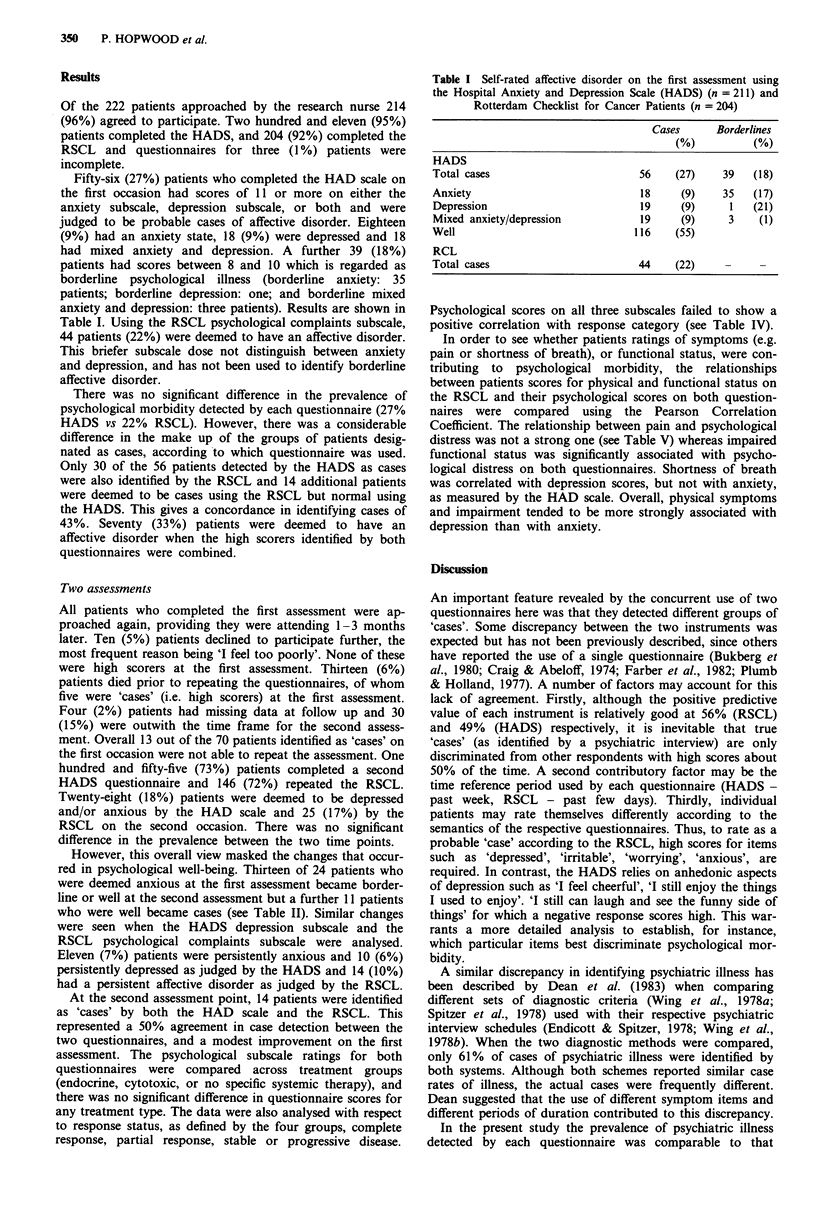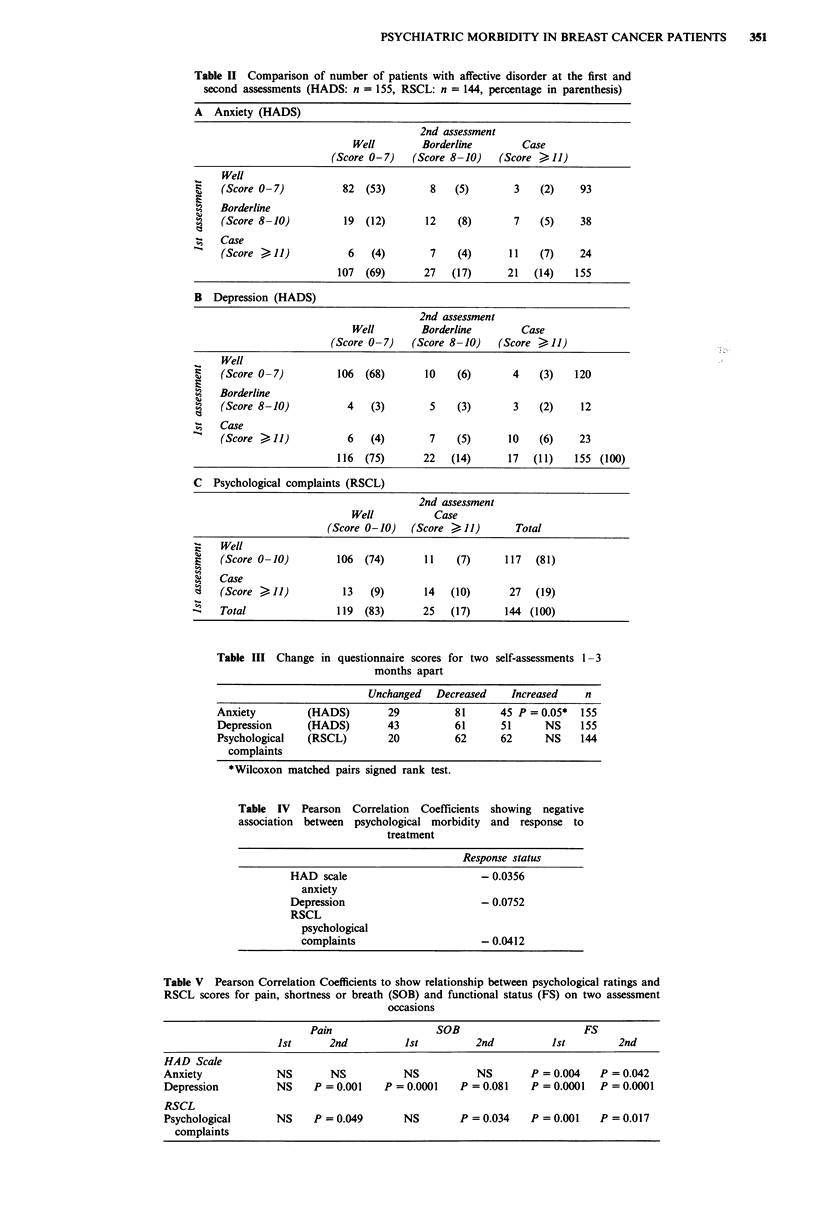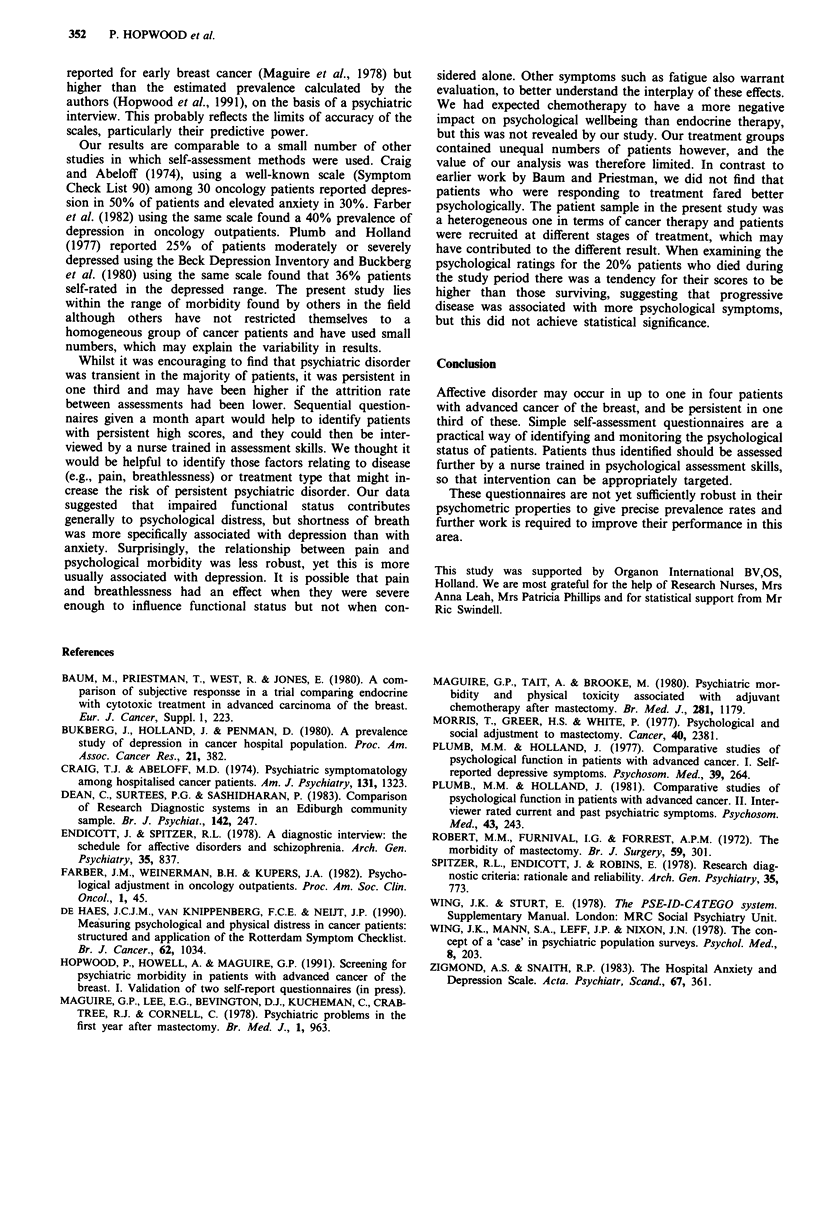# Psychiatric morbidity in patients with advanced cancer of the breast: prevalence measured by two self-rating questionnaires.

**DOI:** 10.1038/bjc.1991.304

**Published:** 1991-08

**Authors:** P. Hopwood, A. Howell, P. Maguire

**Affiliations:** Cancer Research Campaign Psychological Medicine Group, Christie Hospital and Holt Radium Institute, Withington, Manchester, UK.

## Abstract

Two hundred and twenty-two women with advanced cancer of the breast were asked to complete two previously validated self-assessment questionnaires (Hospital Anxiety and Depression Scale (HADS) and the Rotterdam Symptom Checklist (RSCL) in order to determine the prevalence and persistence of affective disorders in this group of patients. Fifty-six (27%) of 211 women who completed the HADS and 33 (22%) of 204 who completed the RSCL rated as probable cases of an anxiety state and/or depressive illness. One hundred and fifty-five patients completed the questionnaires again 1-3 months later. Twenty-one (13%) were persistently anxious or depressed as judged by the HADS compared with 14 (10%) on the RSCL. When both questionnaires were considered together, approximately one third of patients had scores suggestive of an affective disorder and in one third of these it was persistent. Only 30 patients (43% of cases) were detected as 'cases' by both questionnaires and this finding warrants further investigation.


*Br. J. Cancer* (1991), **64**, 349–352 © Macmillan Press Ltd., 1991

### Psychiatric morbidity in patients with advanced cancer of the breast: prevalence measured by two self-rating questionnaires

P. Hopwood[1], A. Howell[2] & P. Maguire[1]

[1]*Cancer Research Campaign Psychological Medicine Group,* [2]*Cancer Research Campaign Department of Medical Oncology, Christie Hospital and Holt Radium Institute, Wilmslow Road, Withington, Manchester M20 9BX, UK.*

**Summary** Two hundred and twenty-two women with advanced cancer of the breast were asked to complete two previously validated self-assessment questionnaires (Hospital Anxiety and Depression Scale (HADS) and the Rotterdam Symptom Checklist (RSCL) in order to determine the prevalence and persistence of affective disorders in this group of patients. Fifty-six (27%) of 211 women who completed the HADS and 33 (22%) of 204 who completed the RSCL rated as probable cases of an anxiety state and/or depressive illness. One hundred and fifty-five patients completed the questionnaires again 1–3 months later. Twenty-one (13%) were persistently anxious or depressed as judged by the HADS compared with 14 (10%) on the RSCL. When both questionnaires were considered together, approximately one third of patients had scores suggestive of an affective disorder and in one third of these it was persistent. Only 30 patients (43% of cases) were detected as 'cases' by both questionnaires and this finding warrants further investigation.

There has been extensive investigation into the psychological sequelae of early breast cancer and its treatment (Maguire *et al.*, 1978; Maguire *et al.*, 1980; Morris *et al.*, 1977; Robert *et al.*, 1972) but there are few data concerning the prevalence of depression and anxiety in patients with advanced disease. There is a need to determine the extent and severity of affective disorder in patients receiving palliative treatment for breast cancer and to find out whether such disorder is transient or warrants intervention.

Plumb and Holland (1981) reported that 20–30% of patients admitted to hospital for the treatment of a variety of advanced cancers developed clinically significant depression and 15% had severe anxiety. In a study of 215 cancer inpatients and outpatients Derogatis and co workers (1983) found 47% met criteria for psychiatric disorder, and of these 13% had a major affective disorder, mainly depression. The majority were described as suffering 'adjustment disorder' that is a less severe form of mood disorder. In a number of other studies (Bukberg *et al.*, 1980; Craig & Abeloff, 1974; Farber *et al.*, 1982; Plumb & Holland, 1977) self report questionnaires have been used to assess psychological morbidity in heterogeneous cancer patient groups. Two such questionnaires were deemed suitable for use for the present study. The Hospital Anxiety and Depression Scale (HADS) (Zigmond & Snaith, 1983) was designed to measure depression and anxiety in medical outpatients and avoids the use of somatic symptoms, which may also be attributed to disease and treatment. It has been shown to be a valid measure of the severity of disorders of mood. The Rotterdam Symptom Checklist (de Haes *et al.*, 1990) was designed as a multidimensional scale for use in quality of life assessment: it contains a subscale of eight items relating to psychological symptoms.

The authors validated these two questionnaires in a series of 81 patients with advanced breast cancer (Hopwood *et al.*, 1991), to establish appropriate cut-off values for the identification of 'cases' of affective disorder. Both questionnaires identified 75% of patients diagnosed as cases by a psychiatric interview. The scales were able to correctly identify one in evey two high scorers as a case and, although this predictive power was high in relation to 'screening' for affective disorder, it will inevitably mean that prevalence rates using self-rating scales are an estimate of morbidity rather than an accurate measure of disorder. Nevertheless, scales such as the HADS and RSCL are being used increasingly to evaluate 'quality of life' in cancer patients and their performance in the measurement of psychological disorder in a homogeneous population of patients warrants evaluation.

The aim of the study reported here was to use these two questionnaires to assess the prevalance of affective disorders in a large outpatient sample of women with advanced breast cancer. A second aim was to determine there was a change in psychological morbidity with time and therefore patients were reassessed after a period of 1–3 months.

Correspondence: P. Hopwood.
Received 16 February 1990; and in revised form 2 April 1991.

### Patients and methods

Two hundred and twenty-two consecutive patients with advanced cancer of the breast attending two specialist Breast Clinics were asked by a trained research nurse to complete the HADS and RSCL. All patients had histologically or radiologically proven advanced cancer of the breast and were attending the clinic in order to be certain that the appropriate palliative treatments were being used to control symptoms and the progression of the disease. One hundred and twenty-seven (57%) patients were receiving endocrine therapy, 48 (21%) were receiving chemotherapy and 47 (22%) were not on specific systemic treatment.

In the validation study a cut-off score of 11 was found to be the optimum value for detecting 'cases' of depression and anxiety using the respective subscales of the HADS. A score of 11 was also the optimum threshold value using the psychological complaints subscale of the RSCL. The prevalence of the affective disorder was measured according to whether a patient's score fell into one of two subgroups: normal, or probable case of affective disorder. Using the HADS scale an additional subgroup was also identified, since a 'borderline affective disorder' group had been defined by its authors using scores between 8 and 10.

The Research Nurse described the purpose of the study and explained to patients how the questionnaires should be completed. All patients were asked to complete the questionnaires again at their next hospital visit. The interval between these assessments ranged between 4 and 12 weeks, and patients were not approached earlier even if they had to return early to the clinic. This decision was made so that assessments would not be burdensome to patients, and so that sufficient time had elapsed to allow for changes in psychological status in the patient sample.

The physical symptom ratings on the RSCL were examined to find out whether key symptoms of advanced breast cancer contributed to psychological morbidity. A similar analysis was carried out for performance status as rated by the patients using the Rotterdam Checklist.

### Results

Of the 222 patients approached by the research nurse 214 (96%) agreed to participate. Two hundred and eleven (95%) patients completed the HADS, and 204 (92%) completed the RSCL and questionnaires for three (1%) patients were incomplete.

Fifty-six (27%) patients who completed the HAD scale on the first occasion had scores of 11 or more on either the anxiety subscale, depression subscale, or both and were judged to be probable cases of affective disorder. Eighteen (9%) had an anxiety state, 18 (9%) were depressed and 18 had mixed anxiety and depression. A further 39 (18%) patients had scores between 8 and 10 which is regarded as borderline psychological illness (borderline anxiety: 35 patients; borderline depression: one; and borderline mixed anxiety and depression: three patients). Results are shown in Table I. Using the RSCL psychological complaints subscale, 44 patients (22%) were deemed to have an affective disorder. This briefer subscale dose not distinguish between anxiety and depression, and has not been used to identify borderline affective disorder.

There was no significant difference in the prevalence of psychological morbidity detected by each questionnaire (27% HADS *vs* 22% RSCL). However, there was a considerable difference in the make up of the groups of patients designated as cases, according to which questionnaire was used. Only 30 of the 56 patients detected by the HADS as cases were also identified by the RSCL and 14 additional patients were deemed to be cases using the RSCL but normal using the HADS. This gives a concordance in identifying cases of 43%. Seventy (33%) patients were deemed to have an affective disorder when the high scorers identified by both questionnaires were combined.

*Two assessments*

All patients who completed the first assessment were approached again, providing they were attending 1–3 months later. Ten (5%) patients declined to participate further, the most frequent reason being 'I feel too poorly'. None of these were high scorers at the first assessment. Thirteen (6%) patients died prior to repeating the questionnaires, of whom five were 'cases' (i.e. high scorers) at the first assessment. Four (2%) patients had missing data at follow up and 30 (15%) were outwith the time frame for the second assessment. Overall 13 out of the 70 patients identified as 'cases' on the first occasion were not able to repeat the assessment. One hundred and fifty-five (73%) patients completed a second HADS questionnaire and 146 (72%) repeated the RSCL. Twenty-eight (18%) patients were deemed to be depressed and/or anxious by the HAD scale and 25 (17%) by the RSCL on the second occasion. There was no significant difference in the prevalence between the two time points.

However, this overall view masked the changes that occurred in psychological well-being. Thirteen of 24 patients who were deemed anxious at the first assessment became borderline or well at the second assessment but a further 11 patients who were well became cases (see Table II). Similar changes were seen when the HADS depression subscale and the RSCL psychological complaints subscale were analysed. Eleven (7%) patients were persistently anxious and 10 (6%) persistently depressed as judged by the HADS and 14 (10%) had a persistent affective disorder as judged by the RSCL.

At the second assessment point, 14 patients were identified as 'cases' by both the HAD scale and the RSCL. This represented a 50% agreement in case detection between the two questionnaires, and a modest improvement on the first assessment. The psychological subscale ratings for both questionnaires were compared across treatment groups (endocrine, cytotoxic, or no specific systemic therapy), and there was no significant difference in questionnaire scores for any treatment type. The data were also analysed with respect to response status, as defined by the four groups, complete response, partial response, stable or progressive disease.

**Table I** Self-rated affective disorder on the first assessment using the Hospital Anxiety and Depression Scale (HADS) ($n = 211$) and Rotterdam Checklist for Cancer Patients ($n = 204$)

| | *Cases* | (%) | *Borderlines* | (%) |
|---|---|---|---|---|
| HADS | | | | |
| Total cases | 56 | (27) | 39 | (18) |
| Anxiety | 18 | (9) | 35 | (17) |
| Depression | 19 | (9) | 1 | (21) |
| Mixed anxiety/depression | 19 | (9) | 3 | (1) |
| Well | 116 | (55) | | |
| RCL | | | | |
| Total cases | 44 | (22) | – | – |

Psychological scores on all three subscales failed to show a positive correlation with response category (see Table IV).

In order to see whether patients ratings of symptoms (e.g. pain or shortness of breath), or functional status, were contributing to psychological morbidity, the relationships between patients scores for physical and functional status on the RSCL and their psychological scores on both questionnaires were compared using the Pearson Correlation Coefficient. The relationship between pain and psychological distress was not a strong one (see Table V) whereas impaired functional status was significantly associated with psychological distress on both questionnaires. Shortness of breath was correlated with depression scores, but not with anxiety, as measured by the HAD scale. Overall, physical symptoms and impairment tended to be more strongly associated with depression than with anxiety.

### Discussion

An important feature revealed by the concurrent use of two questionnaires here was that they detected different groups of 'cases'. Some discrepancy between the two instruments was expected but has not been previously described, since others have reported the use of a single questionnaire (Bukberg *et al.*, 1980; Craig & Abeloff, 1974; Farber *et al.*, 1982; Plumb & Holland, 1977). A number of factors may account for this lack of agreement. Firstly, although the positive predictive value of each instrument is relatively good at 56% (RSCL) and 49% (HADS) respectively, it is inevitable that true 'cases' (as identified by a psychiatric interview) are only discriminated from other respondents with high scores about 50% of the time. A second contributory factor may be the time reference period used by each questionnaire (HADS – past week, RSCL – past few days). Thirdly, individual patients may rate themselves differently according to the semantics of the respective questionnaires. Thus, to rate as a probable 'case' according to the RSCL, high scores for items such as 'depressed', 'irritable', 'worrying', 'anxious', are required. In contrast, the HADS relies on anhedonic aspects of depression such as 'I feel cheerful', 'I still enjoy the things I used to enjoy'. 'I still can laugh and see the funny side of things' for which a negative response scores high. This warrants a more detailed analysis to establish, for instance, which particular items best discriminate psychological morbidity.

A similar discrepancy in identifying psychiatric illness has been described by Dean *et al.* (1983) when comparing different sets of diagnostic criteria (Wing *et al.*, 1978*a*; Spitzer *et al.*, 1978) used with their respective psychiatric interview schedules (Endicott & Spitzer, 1978; Wing *et al.*, 1978*b*). When the two diagnostic methods were compared, only 61% of cases of psychiatric illness were identified by both systems. Although both schemes reported similar case rates of illness, the actual cases were frequently different. Dean suggested that the use of different symptom items and different periods of duration contributed to this discrepancy.

In the present study the prevalence of psychiatric illness detected by each questionnaire was comparable to that

**Table II** Comparison of number of patients with affective disorder at the first and second assessments (HADS: $n = 155$, RSCL: $n = 144$, percentage in parenthesis)

A Anxiety (HADS)

| 1st assessment | 2nd assessment Well (Score 0–7) | 2nd assessment Borderline (Score 8–10) | 2nd assessment Case (Score ⩾11) | |
|---|---|---|---|---|
| *Well (Score 0–7)* | 82 (53) | 8 (5) | 3 (2) | 93 |
| *Borderline (Score 8–10)* | 19 (12) | 12 (8) | 7 (5) | 38 |
| *Case (Score ⩾11)* | 6 (4) | 7 (4) | 11 (7) | 24 |
| | 107 (69) | 27 (17) | 21 (14) | 155 |

B Depression (HADS)

| 1st assessment | 2nd assessment Well (Score 0–7) | 2nd assessment Borderline (Score 8–10) | 2nd assessment Case (Score ⩾11) | |
|---|---|---|---|---|
| *Well (Score 0–7)* | 106 (68) | 10 (6) | 4 (3) | 120 |
| *Borderline (Score 8–10)* | 4 (3) | 5 (3) | 3 (2) | 12 |
| *Case (Score ⩾11)* | 6 (4) | 7 (5) | 10 (6) | 23 |
| | 116 (75) | 22 (14) | 17 (11) | 155 (100) |

C Psychological complaints (RSCL)

| 1st assessment | 2nd assessment Well (Score 0–10) | 2nd assessment Case (Score ⩾11) | Total |
|---|---|---|---|
| *Well (Score 0–10)* | 106 (74) | 11 (7) | 117 (81) |
| *Case (Score ⩾11)* | 13 (9) | 14 (10) | 27 (19) |
| *Total* | 119 (83) | 25 (17) | 144 (100) |

**Table III** Change in questionnaire scores for two self-assessments 1–3 months apart

| | | *Unchanged* | *Decreased* | *Increased* | | *n* |
|---|---|---|---|---|---|---|
| Anxiety | (HADS) | 29 | 81 | 45 | $P = 0.05$* | 155 |
| Depression | (HADS) | 43 | 61 | 51 | NS | 155 |
| Psychological complaints | (RSCL) | 20 | 62 | 62 | NS | 144 |

*Wilcoxon matched pairs signed rank test.

**Table IV** Pearson Correlation Coefficients showing negative association between psychological morbidity and response to treatment

| | *Response status* |
|---|---|
| HAD scale anxiety | – 0.0356 |
| Depression | – 0.0752 |
| RSCL psychological complaints | – 0.0412 |

**Table V** Pearson Correlation Coefficients to show relationship between psychological ratings and RSCL scores for pain, shortness or breath (SOB) and functional status (FS) on two assessment occasions

| | *Pain 1st* | *Pain 2nd* | *SOB 1st* | *SOB 2nd* | *FS 1st* | *FS 2nd* |
|---|---|---|---|---|---|---|
| *HAD Scale* | | | | | | |
| Anxiety | NS | NS | NS | NS | $P = 0.004$ | $P = 0.042$ |
| Depression | NS | $P = 0.001$ | $P = 0.0001$ | $P = 0.081$ | $P = 0.0001$ | $P = 0.0001$ |
| *RSCL* | | | | | | |
| Psychological complaints | NS | $P = 0.049$ | NS | $P = 0.034$ | $P = 0.001$ | $P = 0.017$ |

reported for early breast cancer (Maguire et al., 1978) but higher than the estimated prevalence calculated by the authors (Hopwood et al., 1991), on the basis of a psychiatric interview. This probably reflects the limits of accuracy of the scales, particularly their predictive power.

Our results are comparable to a small number of other studies in which self-assessment methods were used. Craig and Abeloff (1974), using a well-known scale (Symptom Check List 90) among 30 oncology patients reported depression in 50% of patients and elevated anxiety in 30%. Farber et al. (1982) using the same scale found a 40% prevalence of depression in oncology outpatients. Plumb and Holland (1977) reported 25% of patients moderately or severely depressed using the Beck Depression Inventory and Buckberg et al. (1980) using the same scale found that 36% patients self-rated in the depressed range. The present study lies within the range of morbidity found by others in the field although others have not restricted themselves to a homogeneous group of cancer patients and have used small numbers, which may explain the variability in results.

Whilst it was encouraging to find that psychiatric disorder was transient in the majority of patients, it was persistent in one third and may have been higher if the attrition rate between assessments had been lower. Sequential questionnaires given a month apart would help to identify patients with persistent high scores, and they could then be interviewed by a nurse trained in assessment skills. We thought it would be helpful to identify those factors relating to disease (e.g., pain, breathlessness) or treatment type that might increase the risk of persistent psychiatric disorder. Our data suggested that impaired functional status contributes generally to psychological distress, but shortness of breath was more specifically associated with depression than with anxiety. Surprisingly, the relationship between pain and psychological morbidity was less robust, yet this is more usually associated with depression. It is possible that pain and breathlessness had an effect when they were severe enough to influence functional status but not when considered alone. Other symptoms such as fatigue also warrant evaluation, to better understand the interplay of these effects. We had expected chemotherapy to have a more negative impact on psychological wellbeing than endocrine therapy, but this was not revealed by our study. Our treatment groups contained unequal numbers of patients however, and the value of our analysis was therefore limited. In contrast to earlier work by Baum and Priestman, we did not find that patients who were responding to treatment fared better psychologically. The patient sample in the present study was a heterogeneous one in terms of cancer therapy and patients were recruited at different stages of treatment, which may have contributed to the different result. When examining the psychological ratings for the 20% patients who died during the study period there was a tendency for their scores to be higher than those surviving, suggesting that progressive disease was associated with more psychological symptoms, but this did not achieve statistical significance.

### Conclusion

Affective disorder may occur in up to one in four patients with advanced cancer of the breast, and be persistent in one third of these. Simple self-assessment questionnaires are a practical way of identifying and monitoring the psychological status of patients. Patients thus identified should be assessed further by a nurse trained in psychological assessment skills, so that intervention can be appropriately targeted.

These questionnaires are not yet sufficiently robust in their psychometric properties to give precise prevalence rates and further work is required to improve their performance in this area.

This study was supported by Organon International BV,OS, Holland. We are most grateful for the help of Research Nurses, Mrs Anna Leah, Mrs Patricia Phillips and for statistical support from Mr Ric Swindell.

### References

BAUM, M., PRIESTMAN, T., WEST, R. & JONES, E. (1980). A comparison of subjective responsse in a trial comparing endocrine with cytotoxic treatment in advanced carcinoma of the breast. *Eur. J. Cancer*, Suppl. 1, 223.

BUKBERG, J., HOLLAND, J. & PENMAN, D. (1980). A prevalence study of depression in cancer hospital population. *Proc. Am. Assoc. Cancer Res.*, **21**, 382.

CRAIG, T.J. & ABELOFF, M.D. (1974). Psychiatric symptomatology among hospitalised cancer patients. *Am. J. Psychiatry*, **131**, 1323.

DEAN, C., SURTEES, P.G. & SASHIDHARAN, P. (1983). Comparison of Research Diagnostic systems in an Ediburgh community sample. *Br. J. Psychiat.*, **142**, 247.

ENDICOTT, J. & SPITZER, R.L. (1978). A diagnostic interview: the schedule for affective disorders and schizophrenia. *Arch. Gen. Psychiatry*, **35**, 837.

FARBER, J.M., WEINERMAN, B.H. & KUPERS, J.A. (1982). Psychological adjustment in oncology outpatients. *Proc. Am. Soc. Clin. Oncol.*, **1**, 45.

DE HAES, J.C.J.M., VAN KNIPPENBERG, F.C.E. & NEIJT, J.P. (1990). Measuring psychological and physical distress in cancer patients: structured and application of the Rotterdam Symptom Checklist. *Br. J. Cancer.*, **62**, 1034.

HOPWOOD, P., HOWELL, A. & MAGUIRE, G.P. (1991). Screening for psychiatric morbidity in patients with advanced cancer of the breast. I. Validation of two self-report questionnaires (in press).

MAGUIRE, G.P., LEE, E.G., BEVINGTON, D.J., KUCHEMAN, C., CRABTREE, R.J. & CORNELL, C. (1978). Psychiatric problems in the first year after mastectomy. *Br. Med. J.*, **1**, 963.

MAGUIRE, G.P., TAIT, A. & BROOKE, M. (1980). Psychiatric morbidity and physical toxicity associated with adjuvant chemotherapy after mastectomy. *Br. Med. J.*, **281**, 1179.

MORRIS, T., GREER, H.S. & WHITE, P. (1977). Psychological and social adjustment to mastectomy. *Cancer*, **40**, 2381.

PLUMB, M.M. & HOLLAND, J. (1977). Comparative studies of psychological function in patients with advanced cancer. I. Self-reported depressive symptoms. *Psychosom. Med.*, **39**, 264.

PLUMB., M.M. & HOLLAND, J. (1981). Comparative studies of psychological function in patients with advanced cancer. II. Interviewer rated current and past psychiatric symptoms. *Psychosom. Med.*, **43**, 243.

ROBERT, M.M., FURNIVAL, I.G. & FORREST, A.P.M. (1972). The morbidity of mastectomy. *Br. J. Surgery*, **59**, 301.

SPITZER, R.L., ENDICOTT, J. & ROBINS, E. (1978). Research diagnostic criteria: rationale and reliability. *Arch. Gen. Psychiatry*, **35**, 773.

WING, J.K. & STURT, E. (1978). *The PSE-ID-CATEGO system.* Supplementary Manual. London: MRC Social Psychiatry Unit.

WING, J.K., MANN, S.A., LEFF, J.P. & NIXON, J.N. (1978). The concept of a 'case' in psychiatric population surveys. *Psychol. Med.*, **8**, 203.

ZIGMOND, A.S. & SNAITH, R.P. (1983). The Hospital Anxiety and Depression Scale. *Acta. Psychiatr, Scand.*, **67**, 361.